# Cannabis and Vulvodynia Symptoms: A Preliminary Report

**DOI:** 10.26828/cannabis.2020.02.001

**Published:** 2020-07-03

**Authors:** Eliza Barach, Melissa N. Slavin, Mitch Earleywine

**Affiliations:** 1 University at Albany, State University of New York, Albany, NY, USA; 2 Columbia University School of Social Work, New York, NY, USA

**Keywords:** vulvodynia, vulvar pain, cannabis, expectancies, alternative treatment

## Abstract

Medical marijuana has a long history of use as an analgesic for chronic pain disorders, including dyspareunia (pain during intercourse), a hallmark of the rare chronic pain disorder vulvodynia. Many women’s health topics remain under investigated. Few studies address cannabis’s potential to treat vulvodynia symptoms despite their dramatic impact on quality of life. Women who had used cannabis and who reported experiencing vulvodynia symptoms (*N* = 38) completed an online survey assessing symptoms, expectancies regarding cannabis-associated relief from vulvodynia symptoms, cannabis use, and cannabis-related problems. Generally, women expected cannabis to have moderate to large effects on vulvodynia symptoms (*d* = .63-1.19). Nevertheless, women expected greater relief for burning/stabbing pain than for itching and pain associated with tampon insertion, as well greater relief for dyspareunia than for pain associated with tampon insertion. Those whose symptoms were worse expected more relief from cannabis treatment. Expectations of cannabis-induced relief did not increase frequency of use or problems. These data support the idea that further work is warranted, including placebo-controlled randomized clinical trials to rule out any placebo effects and identify potential adverse side effects from a cannabis treatment for vulvodynia.

Vulvodynia, a chronic pain disorder affecting the vulva, persists for at least three months and lacks a clear identifiable cause (Borstein et al., 2016). The disorder affects up to 28% of women in their lifetime (Groysman, 2010). The pain can be localized, affecting a specific area in the vulva (e.g., the vestibule), generalized (affecting the vulva as a whole), or mixed (both localized and generalized pain; Borstein et al., 2016). Patients often describe the pain as ‘knife-like’, burning, rawness, or itching (Sadownik, 2014). Both sexual (e.g., vaginal penetration) and non-sexual contact (e.g., clothing, tampon insertion) can elicit pain, but symptoms also appear spontaneously. Mixed conditions also appear where both physical contact and the absence of contact can elicit pain. The pain might arise from the first attempts of vaginal penetration (i.e., primary vulvodynia) or appear after a period of pain-free sexual intercourse (i.e., secondary vulvodynia; Bornstein et al., 2016). The exact cause of vulvodynia remains unknown, but is likely multifactorial (Sadownik, 2014) with established links to inflammation (Falsetta et al., 2015) and neuroproliferation of nociceptors (Bohm-Starke, Hilliges, Falconer, & Rylander, 1999).

## Current Treatment Options for Vulvodynia

Proposed treatments include reducing potential irritants, administering topical analgesics, injections, oral medications, and surgery. Reducing irritants often includes changing laundry detergent, switching to cotton underwear, and avoiding tight clothing. Topical analgesics often include regular applications of lidocaine, especially prior to sexual contact, but many patients find this approach increases irritation for themselves or their male partners, and might not surpass placebo (Foster et al., 2010). Similarly, injections of Botulinum toxin A have alleviated self-reported pain in one study (Hansen, Guildberg, & Meinert, 2019), but failed to surpass placebo in another (Petersen, Giraldi, Lundvan & Kristensen, 2009). Health professionals have turned to prescription tricyclic antidepressants (TCAs), Serotonin Reuptake Inhibitors (SSRIs), and anticonvulsants, (e.g., gabapentin) with some success (Leo, 2013; Leo & Dewani, 2013), but attempts at replication show no advantage for either approach over placebo (Brown, Bachmann, Wan, & Foster, 2018; Foster et al., 2010). The side-effects of some of these medications decrease adherence to the treatments too. In summary, none of these treatment options have uniform successes in reducing vulvodynia pain, with many failing to outperform reasonable placebos (Miranda, Soriano, Silveira, & Vale, 2018).

Finally, health professionals have recommended a vestibulectomy (e.g., partial or full removal of the vulvar vestibule; Haefner et al., 2005) after more conservative treatments have failed. The surgery can improve localized provoked vulvodynia, but appears less effective for generalized vulvodynia (Falsetta, Foster, Bonham, & Phipps, 2017). Potential complications include infection, increased pain, reduction in lubrication and sensitive scar tissue (Tommola, Unkila-Kallio, & Paavonen, 2010). The lack of randomized clinical trials (RCTs) prevents reasonable estimates of rates of true success or complications of this procedure (Stockdale & Lawson, 2014). Many women are reluctant to turn to surgery for this ailment. Alternative treatments with better outcome and side-effect profiles would be a dramatic improvement.

## Vulvodynia and Cannabis

Historically, medical marijuana is most commonly used for chronic pain (Parker, 2017). Anecdotal reports suggest that marijuana can treat vulvodynia. Cannabis offers significant analgesic effects with few side effects for a wide range of chronic pain conditions, including neuropathic pain, fibromyalgia, rheumatoid arthritis, and mixed chronic pain (Lynch & Campbell, 2011; Lynch & Ware, 2015). Furthermore, cannabis exhibits potential anti-inflammatory properties (Blake, Robson, Ho, Jubb, & McCabe, 2006) and at low doses can effectively treat chronic pain while avoiding the psychoactive side effects (Wilsey et al., 2013). Dyspareunia, a hallmark symptom of vulvodynia, decreases when patients consume marijuana prior to intercourse (Lynn, López, Miller, Thompson, & Campian, 2019). Marijuana’s success with neuropathic and inflammatory pain (as well as dyspareunia) support its potential efficacy for vulvodynia.

## Cannabis Treatment Expectancies: Links to Cannabis Use and Associated Problems

Expectancies about a host of substances influence their subjective experience and subsequent use. Cannabis use often increases as expectancies for positive effects increase. Those who anticipate more enhanced social interactions, improved sexual functioning, or greater relaxation use cannabis more often or in greater amounts (e.g. Metrik et al., 2009; Schafer & Brown, 1991). Those who think cannabis will improve their symptoms are likely to consume cannabis more frequently or in greater quantities as well. For example, symptom severity for PTSD (Earleywine & Bolles, 2014), menopause (Slavin, Farmer, & Earleywine, 2016), and PMS/PMDD (Slavin, Barach, Farmer, Luba, & Earleywine, 2017) correlated with individuals’ cannabis use. Expectancies of cannabis-induced relief mediated the associations between symptom severity and use in these examples as well. Comparable symptom-specific expectancies might exist for women who use cannabis for vulvodynia relief, and their consumption might covary with these expectancies.

Nevertheless, positive expectancies need not correlate with cannabis-related problems. For example, menopausal women and women suffering from PMS/PMDD reported expecting cannabis to relieve these symptoms, but the expectancies varied inversely with problems (Slavin et al., 2017; Slavin et al., 2016). In contrast, regular and heavy users expectations of global negative effects did increase with problems (Beraha, Cousijn, Hermanides, Goudriaan, & Wiers, 2013). Ideally, expectations of relief from vulvodynia would not lead to inordinate or problematic use.

## Current Study

Because cannabis improves many different chronic pain conditions (Lynch & Campbell, 2011; Lynch & Ware, 2015) and reduces dyspareunia (Lynn et al., 2019), we hypothesized that participants would expect cannabis to alleviate vulvodynia symptoms. In addition, we examined the relation between cannabis treatment expectancies, monthly cannabis use and vulvodynia pain symptom severity as well as their impact on cannabis-related problems. In order for cannabis to be considered a proper alternative treatment for vulvodynia, it is imperative that the potential medicinal benefits outweigh any associated increase in problems.

## METHOD

### Participants

Women who reported vulvodynia symptoms as well as lifetime cannabis use (*N* = 38) completed an online survey on symptoms, expectancies regarding cannabis-associated relief from vulvodynia symptoms, cannabis use, and cannabis related problems. Participants responded to a Facebook or Vulvodynia support Forum blog post advertisement to complete an internet survey on cannabis use and vulvodynia symptoms. To target women with vulvodynia the advertisement was posted monthly in different Facebook groups that served as vulvodynia support groups as well as in the Vulvodynia support forum webpage. The advertisement stated that participants experiencing vulvodynia and have used cannabis before are eligible to participate in an online research study. Participants were informed that they would be entered into a raffle to win a free vaporizer if selected. Participants had the option to send their email address and a secret number to be eligible for the prize. All procedures were in line with and approved by the local Institutional Review Board.

### Measures

#### Demographics.

Participants reported age, race, ethnicity, education and recent cannabis use. In addition, we asked about disorders frequently comorbid with vulvodynia, including fibromyalgia (Sadownik, 2000), interstitial cystitis (Rueda, 1986), and irritable bowel syndrome (Sadownik, 2000). We also asked about rates of depression/anxiety because of suggested links between depression/anxiety and vulvodynia (Khander et al., 2011) and because living with vulvodynia can have a marked influence on the quality of life (Ponte, Klemperer, Sahay, & Chren, 2009). See Table 1 for participant demographics.

#### Vulvodynia Symptoms.

We assessed Vulvodynia symptoms for generalized vulvodynia and localized vulvodynia as well as for provoked and spontaneous vulvodynia based on common patient self-reported descriptions of vulvodynia symptoms/pain (e.g., Reed et al., 2012; Sutton, Bachmann, Arnold, Rhoads, & Rosen, 2008). The 12 items addressed: (1) vulvar burning, (2) vulvar soreness, (3) vulvar stinging (4) vulvar rawness, (5) vulvar throbbing, (6) vulvar stabbing or sharp pain (7) vulvar itching (8) Dyspareunia, (9) pain with tampon insertion (10) vulvar pain from prolonged sitting (11) vulvar pain from tight fitting pants (12) vulvar pain while exercising. Participants were asked to rate the severity of each symptom on a Likert scale from “none (0)” to “extremely severe (5)”. All symptoms were endorsed by most participants (See Table 1). Cronbach’s Alpha for the total symptoms scale was .878.

#### Expectancies of cannabis-induced changes in Vulvodynia Symptoms.

This scale was adapted from the scale used to assess vulvodynia symptoms and queried participants on their expectancies of how cannabis helps each of these symptoms. Individuals were asked to rate how cannabis makes each of the symptoms feel on a five-point Likert scale ranging from ‘extremely worse (−2)’ to ‘extremely better’. Scores greater than zero on this scale are indicative of more positive expectancies toward cannabis treating vulvodynia symptoms; scores less than zero are indicative of no expectancies of cannabis treating symptoms or expectancies of cannabis worsening symptoms. Cronbach’s Alpha for the total expectancies scale was .916.

#### Cannabis use per month.

Participants reported how many days they used cannabis in the previous month, from zero to 31 days. Average monthly usage was 17.26 days.

#### Cannabis-related problems.

Participants completed the Marijuana Problems Scale by rating 19 items from 0 (none) to 5 (a serious problem) based on the previous 90 days. Items included: (1) problems with partner, (2) problems in family, (3) neglect of family, (4) problems with friends, (5) missing days at work or school, (6) losing a job, (7) lowering productivity, (8) medical issues, (9) withdrawal symptoms, (10) blackouts or flashbacks, (11) memory loss, (12) difficulty sleeping, (13) financial difficulties, (14) legal problems, (15) low energy levels, (16) feeling bad about use, (17) lowered self-esteem, (18) procrastination, and (19) lack of self-confidence. Cronbach’s alpha was .863.

### Data Analysis

We performed a series of one-sample *t*-tests to see if mean expected relief differed from zero for each expectancy. We then performed paired *t*-tests to determine whether women expected greater cannabis-associated relief for some vulvodynia symptoms than others. Due to the number of analyses we used a modified Bonferroni approach to balance power and Type I error (Wilcox, 2013). Each category of analyses received a family-wise error rate of *p* < .05 (two-tailed). For the 12 analyses addressing if the expectancy exceeded zero, corrected p values were set to *p* < .004 (.05/12). Lastly, we compared correlations between frequency of cannabis-usage, cannabis-associated problems, vulvodynia symptoms and cannabis-associated relief expectancies. Given the rarity of the disorder, sample sizes in vulvodynia research are frequently small. Power analysis revealed that with *N* = 38, and alpha at .05 (two-tailed), we have power of 0.80 to detect a Pearson's R of 0.40. *T*-tests to assess significant difference from 0 under the same conditions could detect effects of *d* = 0.47 or larger. Paired t-tests for comparing symptom expectancies could detect *d* = 0.46 or larger (Faul, Erdfelder, Buchner, & Lang, 2009). Given the limited amount of research on this topic and the social desirability bias associated with these symptoms (and cannabis consumption), we were eager to examine even a small sample in an effort to discover if further work on this topic appeared justified.

## RESULTS

### Expectancies of Vulvodynia Symptom Relief

One sample *t*-tests revealed that all expectancies were significantly different from 0 (see Table 2 for means, *t*s, *p*s and the effect size *d*). Paired *t*-tests revealed that participants endorsed greater cannabis-induced relief for sharp/stabbing pain compared to itching (*t*(33) = 3.27, *p* = .002) and tampon insertion (*t*(33) = 3.78, *p* =.001). In addition, participants reported greater cannabis-induced relief for dyspareunia compared to tampon insertion (*t*(34) = 3.43, *p* = .002). The remaining paired *t*-tests did not reveal any difference among the symptoms for expectancies of cannabis-associated relief (all *ps* > .004).

### Bivariate Correlations Among Expectancies, Use, and Problems

Correlations among the severity of symptoms, expectancies for cannabis-induced relief, and cannabis problems appear in Table 3. As symptom severity increased, expectancies regarding cannabis-induced relief increased. No other correlations were significant.

## DISCUSSION

Given the diverse range of efficacy and frequent negative side-effects for treatment for vulvodynia, we examined self-reported responses to cannabis in a sample of women suffering from the disorder. Women reported the severity of their symptoms, expectancies regarding cannabis-induced symptom relief, frequency of cannabis consumption, and cannabis-related problems. Women endorsed cannabis-induced relief for all symptoms. Women expected cannabis to reduce dyspareunia—a hallmark of vulvodynia. These results suggest that cannabis might serve as a promising alternative treatment to add to the arsenal of potential interventions.

In addition, participants expected more cannabis-induced relief for sharp/stabbing symptoms compared to itching and tampon insertion as well as greater expectancies of relief for dyspareunia compared to tampon insertion. These results suggest that cannabis might be better suited for some vulvodynia symptoms, specifically pain. For the remaining symptoms there was no difference in the endorsement of cannabis-relief, suggesting that women expect marijuana to equally help the remaining symptoms associated with vulvodynia. These results are consistent with other work on medical cannabis and chronic pain (Parker, 2017; Lynch & Campbell, 2011; Lynch & Ware, 2015). The absence of a significant link with cannabis-related problems might allay concerns about the development of negative consequences from this treatment approach. Although the current data are preliminary, further work on this topic appears justified.

### Limitations

The current study carries important limitations related to sample size, self-report bias and the scope of the assessed constructs. These data appear to be the first to address this aspect of cannabis expectancies in women’s health. The current sample is small (*N* = 38), but in light of the relative infrequency of vulvodynia (Harlow et al., 2014; Reed et al., 2012), the prevalence of cannabis use, and the low rate of volunteering for cannabis research (Mian, Altman, & Earleywine, 2019), the target population proved relatively small despite cannabis’s potential as a treatment. The current sample is actually larger than many in the published literature on vulvodynia, and these results appear to justify further work on this topic. The potential for self-report bias, particularly given this combination of sensitive topics (drug use and symptoms that affect the genitals), seems high, but the anonymity of internet responding might have helped keep systematic over- or under-reporting to a minimum. Lastly, to avoid increasing the respondent burden, we limited our questionnaire to the items most relevant to participants’ vulvodynia symptoms, marijuana consumption and their expectancies regarding whether they believed marijuana could help reduce their vulvodynia symptoms. Nevertheless, future work should consider additional items regarding participant’s source of obtaining marijuana, the timing of consumption relative to the onset of symptoms, additional drug use (e.g., alcohol consumption), as well as their current vulvodynia treatments. Future work also could attempt to recruit women from vulvovaginal clinics or doctor’s offices that specialize in the treatment of vulvodynia. Alternatively, researchers might cast a wide net among large samples of women and ask them about individual symptoms and their expectations of cannabis-induced relief. Prohibition also limits the number of women who might know about the impact of cannabis on vulvodynia. Ideally, a placebo-controlled randomized clinical trial with cannabis that uses a daily diary approach to track use and symptoms would help reveal the potential for efficacy. Given the current assessment of expectations for cannabis-induced relief, such a trial appears justified.

## Figures and Tables

**Table 1.** Characteristics of Study Sample

Characteristic	*n*	%
Age	34	89.5
19-29	19	50.0
30-39	6	15.8
40-39	4	10.5
50-59	4	10.5
60+	1	2.6
Race/ethnicity	38	100.0
Caucasian	36	94.7
Asian	1	2.6
Hispanic/Latino	1	2.6
Comorbid Illness/Disorders (items not mutually exclusive)	38	100.0
Fibromyalgia	3	7.9
interstitial cystitis	4	10.5
Irritable Bowel Syndrome	6	15.8
Depression/Anxiety	24	63.2
None	12	31.6
Education	38	100.0
Some high school	1	2.6
Finished high school/GED	4	10.5
Some college	11	28.9
Associates Degree	2	5.3
Bachelors Degree	9	23.7
Some graduate training	4	10.5
Advanced degree	7	18.4
Recent Cannabis Use (Items not mutually exclusive)	38	100.0
Past Year	38	100.0
Past Month	34	89.5
Past Week	30	78.9
Vulvodynia Symptoms (items not mutually exclusive)	38	100.0
Vulvar Burning	36	94.7
Vulvar Soreness	33	86.8
Vulvar Stinging	32	84.2
Vulvar Rawness	31	81.6
Vulvar Throbbing	25	65.8
Stabbing or Sharp pain	32	84.2
Vulvar itching	27	71.1
Dyspareunia	37	97.4
Tampon insertion	29	76.3
Vulvar Pain from prolonged sitting	29	76.3
Vulvar Pain from tight fitting pants	31	81.6
Vulvar Pain while exercising	32	84.2

**Table 2.** Mean Expectancies for Vulvodynia Symptoms

Vulvodynia Symptom	Mean (SD)	Significance (2- tailed).	Effect Size (d)
Sharp/Stabbing	.64 (.54)	*t*(35) = 7.06, *p* <.001	1.19
Dyspareunia	.62 (.49)	*t*(36) = 7.69, *p* <.001	1.24
Soreness	.54(.56)	*t*(36) = 5.90, *p* <.001	0.96
Sitting	.53 (.51)	*t*(33) = 6.09, *p* <.001	1.03
Burning	.50 (.60)	*t*(37) = 5.10, *p* <.001	0.83
Stinging	.50 (.56)	*t*(37) =5.53 , *p* <.001	0.89
Throbbing	.47 (.56)	*t*(33) = 4.87, *p* <.001	0.84
Rawness	.42 (.55)	*t*(35) = 4.51, *p* <.001	0.76
Exercise	.35 (.49)	*t*(33) = 4.24, *p* <.001	0.71
Tight Pants	.34 (.59)	*t*(34) = 4.24 , *p* = .002	0.58
Itching	.31(.47)	*t*(34) = 3.95 , *p* <.001	0.70
Tampon Insertion	.29 (.46)	*t*(34) = 3.69 , *p* = .001	0.63

*Note.* Expectancies are ordered from highest (most expected cannabis-induced relief) to lowest.

**Table 3.** Table of Correlations

		Mean (SD)	1	2	3
1	Monthly Use	17.26 (11.62)			
2	Symptoms	24.47 (10.11)	*r* = .22, *p* = .187		
3	Expectancies	5.25 (4.57)	*r* = .20, *p* = .232	***r* = .40, *p* = .013**	
4	Problems	6.64 (7.83)	*r* = .20, *p* = .240	*r* = .17, *p* = .299	*r* = −.04, *p* = .791

*Note.* The degrees of freedom are 36 and significance is 2-tailed. Significant correlations are shown in bold. Monthly use = average number of days of cannabis use per month; Symptoms= Severity of Vulvodynia symptoms; Expectancies = expected cannabis-induced relief of Vulvodynia symptoms; Problems= cannabis-related problems.
